# Unveiling the immune landscape and microenvironmental architecture of IgA nephropathy using single‐cell imaging mass cytometry

**DOI:** 10.1002/cti2.70088

**Published:** 2026-03-13

**Authors:** Xing Chen, Long Liu, Lijing Yao, Jianxin Shen, Hengyuan Zhang, Leping Shao, Xingxing Yu, Zhijuan Lin, Lu Zhang

**Affiliations:** ^1^ Department of Nephrology (Fujian Provincial Clinical Research Center for Glomerular Nephritis), The First Affiliated Hospital of Xiamen University, School of Medicine Xiamen University Xiamen China; ^2^ Department of Hematology, The First Affiliated Hospital of Xiamen University and Institute of Hematology, School of Medicine Xiamen University Xiamen China; ^3^ Key Laboratory of Xiamen for Diagnosis and Treatment of Hematological Malignancy Xiamen China; ^4^ The School of Clinical Medicine Fujian Medical University Fujian China

**Keywords:** IgA nephropathy, Imaging mass cytometry, Immune microenvironment

## Abstract

**Objective:**

The heterogeneity in the pathology of IgA nephropathy (IgAN) represents a significant gap in current research, emphasising the need for individualised treatment strategies. The role of the glomerular microenvironment in driving IgAN heterogeneity remains largely unknown.

**Methods:**

This study employed Imaging Mass Cytometry (IMC) to analyse 34 primary IgAN renal biopsies. A panel of 35 protein markers was used to identify and characterise 17 cell clusters.

**Results:**

Immune cells composed of 9.64% of the glomerular cell population, with macrophages being the predominant subset. While there was no significant difference in the overall percentage of immune cells across glomeruli exhibiting different pathological features, including mesangial proliferation, global sclerosis, segmental sclerosis and crescents, the infiltration pattern of these cells was distinct. Neighbourhood analysis revealed that the interaction mode of M1‐like macrophages varied significantly among these four glomerular pathology types. Severe IgAN (Lee grades IV–V) was associated with a lower glomerular M1/M2‐like macrophage infiltration ratio compared to mild IgAN (Lee grade III). The M1/M2‐like macrophage ratio positively correlated with the estimated glomerular filtration rate (eGFR), while M2‐like macrophages correlated with proteinuria, and T cells were associated with haematuria.

**Conclusions:**

Our study findings underscore that glomerular immune cell subtypes and their interaction patterns critically influence pathological and clinical outcomes, suggesting a new potential direction for molecular therapeutic strategies targeting the IgAN microenvironment.

## Graphical abstract

In IgA nephropathy (IgAN), innate and adaptive immune cells display distinct spatial compartmentalisation, with macrophages predominating within glomeruli and CD4^+^ T cells constituting the major immune population in inflammatory foci. Glomeruli with distinct pathological morphologies exhibit morphology‐dependent immune infiltration patterns and differential intensities of intraglomerular cell–cell interactions, reflecting heterogeneous immune microenvironments. Clinically, a higher M1/M2‐like macrophage ratio is associated with preserved glomerular filtration rate, whereas M2‐like macrophages correlate with proteinuria and T‐cell infiltration with haematuria, highlighting immune cell‐specific links to renal injury phenotypes.

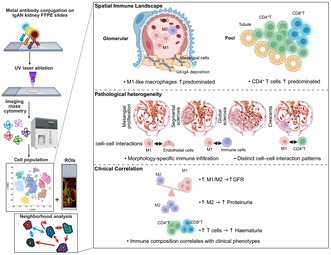

## Introduction

IgA nephropathy (IgAN) represents the predominant form of primary glomerulonephritis globally and is a key contributor to chronic kidney disease and end‐stage renal disease (ESRD).^1^ The prevalence of IgAN shows a distinct geographic pattern, with the highest rates reported in the East, especially in China, accounting for 45–58% of primary glomerulonephritis cases. In contrast, its prevalence is notably lower in Europe.^2^ Diagnosis of IgAN is confirmed through kidney biopsy findings, which reveal the characteristic deposition of immunoglobulin A1 (IgA1) in the glomerular mesangium.^3^, ^4^ The clinical course of IgAN is highly heterogeneous, with symptoms varying from asymptomatic microscopic haematuria or periodic macroscopic haematuria with preserved renal function to substantial proteinuria or even rapidly progressive glomerulonephritis.^5^ Although the overall survival of affected individuals may be slightly diminished, this outcome is largely dependent on the progression of the disease to renal failure.^6^ While the precise mechanisms of IgAN are not yet fully elucidated, a multi‐hit hypothesis has been put forward.^7^ According to this model, irregular O‐glycosylation of IgA1 results in the generation of galactose‐deficient IgA1 (Gd‐IgA1).^8^ These molecules then form circulating immune complexes with autoantibodies, primarily IgG, IgA, or IgM, that bind to polymeric Gd‐IgA1, leading to their accumulation in the glomerular mesangium. This process initiates a series of responses, including the proliferation of mesangial cells, the build‐up of glomerular matrix, infiltration by immune cells and activation of complement pathways in the kidney.^9^ Ultimately, these events result in progressive kidney damage.

Based on findings from genome‐wide association studies (GWAS), it is understood that the dysregulation of both innate and adaptive immunity is a fundamental driver of IgAN.^10^ However, existing single‐cell RNA sequencing studies, limited by sample size and the number of lysed cells, provide only limited quantitative data on detectable cell populations and the immune molecular composition of IgAN biopsies.^11^, ^12^, ^13^ The pathological heterogeneity observed among glomeruli in conditions such as IgAN makes it challenging to accurately identify the specific location of immune cells as either glomerular or interstitial infiltrates. This heterogeneity necessitates the use of advanced research techniques. However, it also underscores the significant potential for developing targeted immunomodulatory therapies that can specifically address this heterogeneity.^10^ Despite their use, current immunosuppressive treatments often exhibit insufficient efficacy and safety for routine use in IgAN.^14^, ^15^ A deeper understanding of the immune infiltrates driving the disease's heterogeneity could provide clearer guidance for developing more effective immunotherapies.

Imaging mass cytometry (IMC) is an innovative technology that facilitates the simultaneous *in situ* visualisation of over 30 protein markers on a single formalin‐fixed, paraffin‐embedded (FFPE) tissue section. Tissues hybridised with cocktails of rare metal isotope‐conjugated antibodies are laser‐ablated with 1‐μm resolution and analysed via mass spectrometry.^16^, ^17^ IMC provides two significant advantages: It preserves spatial context, enabling the identification of cellular interactions, compositions and protein distributions^18^, ^19^; and the absence of metal tags in human organs results in low background noise and high precision, facilitating detailed phenotypic analysis. These advantages make IMC a promising tool for providing novel insights into the patterns of immune dysregulation and activity in IgAN. Based on these advantages, we designed a study using IMC to explore spatial immune cell infiltration that contributes to the pathological heterogeneity in primary IgAN biopsy samples. Our aim was to identify differences in immune cell proportions and modes of interaction that underlie the diverse manifestations of IgAN and could serve as indicators for targeted treatments.

## Results

### IMC procedure for IgAN biopsy samples

#### Cohort design

All 34 donors were clinically diagnosed with primary IgAN and pathologically classified according to the Oxford classification as M1ExSxTx‐Cx (Mesangial hypercellularity score = 1, Endocapillary hypercellularity = 0 or 1, Segmental glomerulosclerosis with or without the presence of an adhesion = 0 or 1, Tubular atrophy/interstitial fibrosis = 0–2, Cellular and/or fibro‐cellular crescents = 0–2), Lee grades III–V. The cohort consisted of 17 females (50%), with a mean age of 34 years (range, 20–55) (Supplementary table 1). All patients received the maximally tolerated dose of Angiotensin‐Converting enzyme inhibitors (ACEi) or angiotensin II receptor blockers (ARB), and/or glucocorticoids, in accordance with the KDIGO 2021 Guidelines.^15^ Patients were followed up for 24 months after biopsy (Supplementary figures 1a and 1b).

#### Antibody panel design

Antibody selection focussed on three functional categories in limited seats (Supplementary figure 2): stromal cell markers to delineate kidney structures, immune cell markers to identify immune cells and cell signalling markers associated with proliferation (such as Ki67, phospho‐STAT3) or specific IgAN mechanisms: JCHAIN^13^ (joining chain of multimeric IgA and IgM), CD71 (Transferrin receptor 1,TfR1)^20^, ^21^ and CD161 (marker of mucosal‐associated invariant T cells, MAIT)^22^ (Supplementary figure 3).

**Figure 1:**
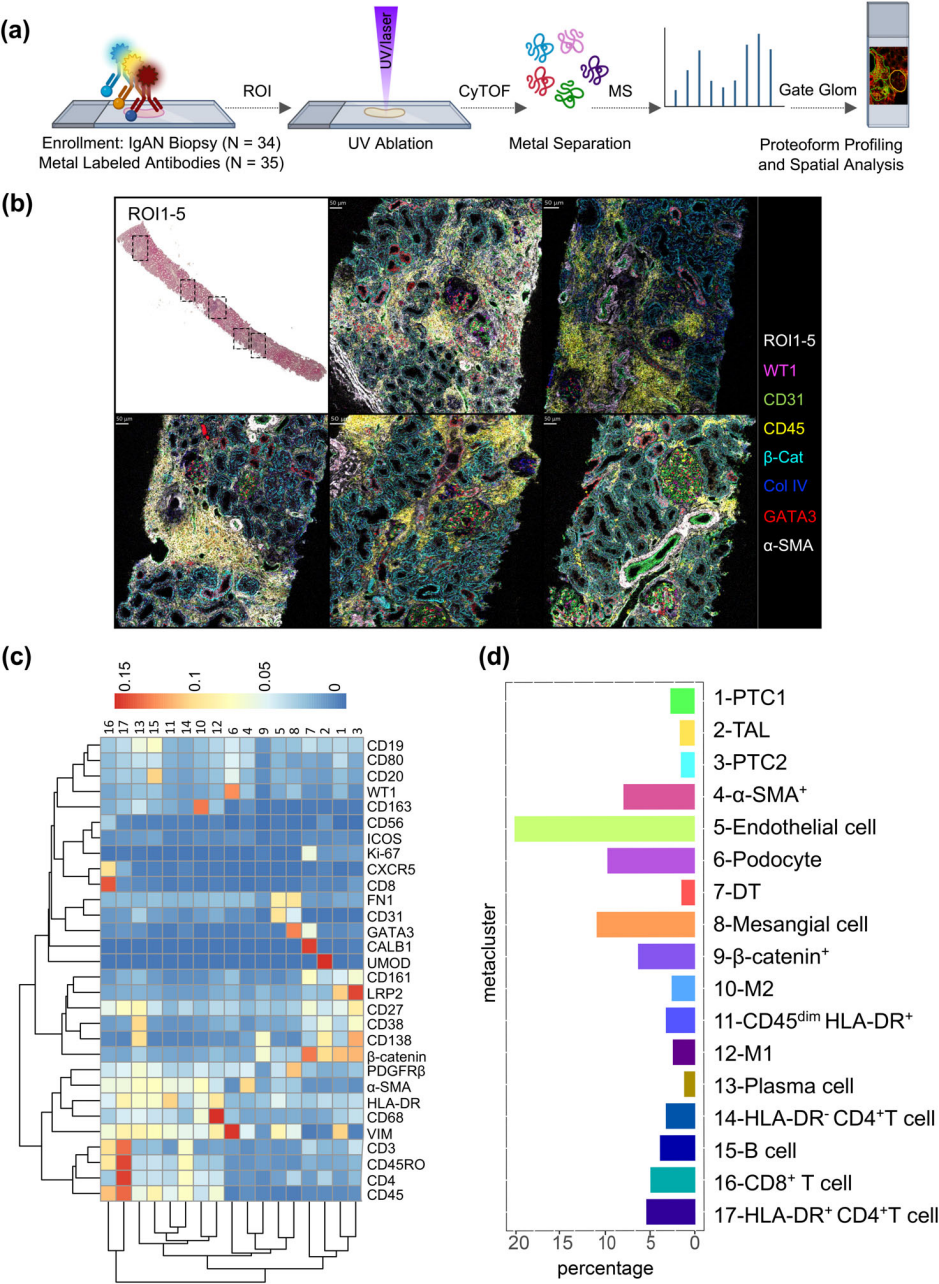
The procedure for applying IMC to IgAN biopsy samples. **(a)** General workflow. Patient Inclusion Criteria: Refer to Methods (*N* = 34), Antibody selection: Refer to Table 1 (*N* = 35); CyTOF Experiment and Mass Spectrometry Identification: regions of interest (ROI) selection composed of 2–8 sections of 600 μm × 600 μm per slide, encompassing 0–5 glomeruli and foci of inflammatory infiltrates. The UV Laser isolates metal‐tagged proteins in ROIs, Mass spectrometry identifies protein markers and enables cell‐type clustering. Analytical Procedures: Further analysis based on spatial structure (glomeruli/foci) and pathological type. **(b)** Actual image showing the selection of 5 ROIs from HE staining of a single biopsy, along with their overall appearance in the MCD viewer. The image displays the basement membrane (Collagen IV, blue), endothelial cells (CD31, green), podocytes (WT1, magenta), mesangial cells (GATA3, red), all tubules (β‐catenin, teal), pan‐immune cells (CD45, yellow), smooth muscle and interstitial cells (α‐SMA, white). The depicted image corresponds to the patient's pathological ID 778 diagnosis as Lee IV (M1E1S1T0C1). Scale bar = 50 μm. **(c)** Cell population heatmap of all clusters identified by the 35‐antibody panel from all the ROIs. The X‐axis represents the cluster number, and the Y‐axis represents the cell marker. A total of 17 clusters were defined across all selected ROI sections. **(d)** Percentages of the 17 cell clusters: the most abundant cell population is the endothelial cell, followed by podocytes and mesangial cells. MS: Mass Spectrometry; CyTOF: Cytometry by Time‐of‐Flight.

After staining with the specified antibody panel, the tissue slides were analysed using IMC based on cytometry by time‐of‐flight (CyTOF) mass spectrometry (Figure 1a). The region of interest (ROI) gating and analytical strategy was based on spatial structure (glomeruli/foci of inflammatory infiltrates) and glomerular pathological features (Figure 1b, Supplementary figure 4). Following this workflow, a total of 302 intact glomeruli and 130 inflammatory foci were selected and analysed across all ROIs.

### Characterisation of 17 cell types in IgAN kidneys

Utilising unsupervised manifold approximation and projection (UMAP) on the 35‐protein expression profiles of all segmented cells, we identified 17 distinct cell clusters (Figures 1c and d). These clusters encompassed resident and infiltrating cell populations. The resident cell types composed of proximal tubular cells (PTC, Clusters 1 and 3, β‐catenin^+^ LRP2^+^), distal tubules (DT, Cluster 7, β‐catenin^+^ CALB1^+^), thick ascending limb of Henle's loop (TAL, Cluster 2, β‐catenin^+^ UMOD^+^), endothelial cells (Cluster 5, CD31^+^), mesangial cells (Cluster 8, PDGFRβ^+^ GATA3^+^), α‐SMA expressing cells (Cluster 4) and podocytes (Cluster 6, WT1^+^ VIM^+^). The immune cell populations encompassed M1‐like macrophages (Clusters 12, CD68^+^ CD80^+^ CD163^−^), M2‐like macrophages (Clusters 10, CD163^+^), various T‐cell subsets, including HLA‐DR^−^ CD4^+^ T cells (Cluster 14), HLA‐DR^+^ CD4^+^ T cells (Cluster 17) and CD8^+^ T cells (cluster 16), plasma cells and B cells (Cluster 13, CD38^+^ CD138^+^ and 15, CD19^+^ CD20^+^) and other immune cells (Cluster 11, CD45^dim^ HLA‐DR^+^). Initial cell type categorisation was executed using a computational algorithm. This automated assignment was then rigorously validated through expert manual curation to confirm that each designated cell population demonstrated the expected immunohistochemical profile within the ROIs.

### Distinct spatial distribution of innate and adaptive immune cells in glomeruli and foci of IgAN kidneys

**Figure 2:**
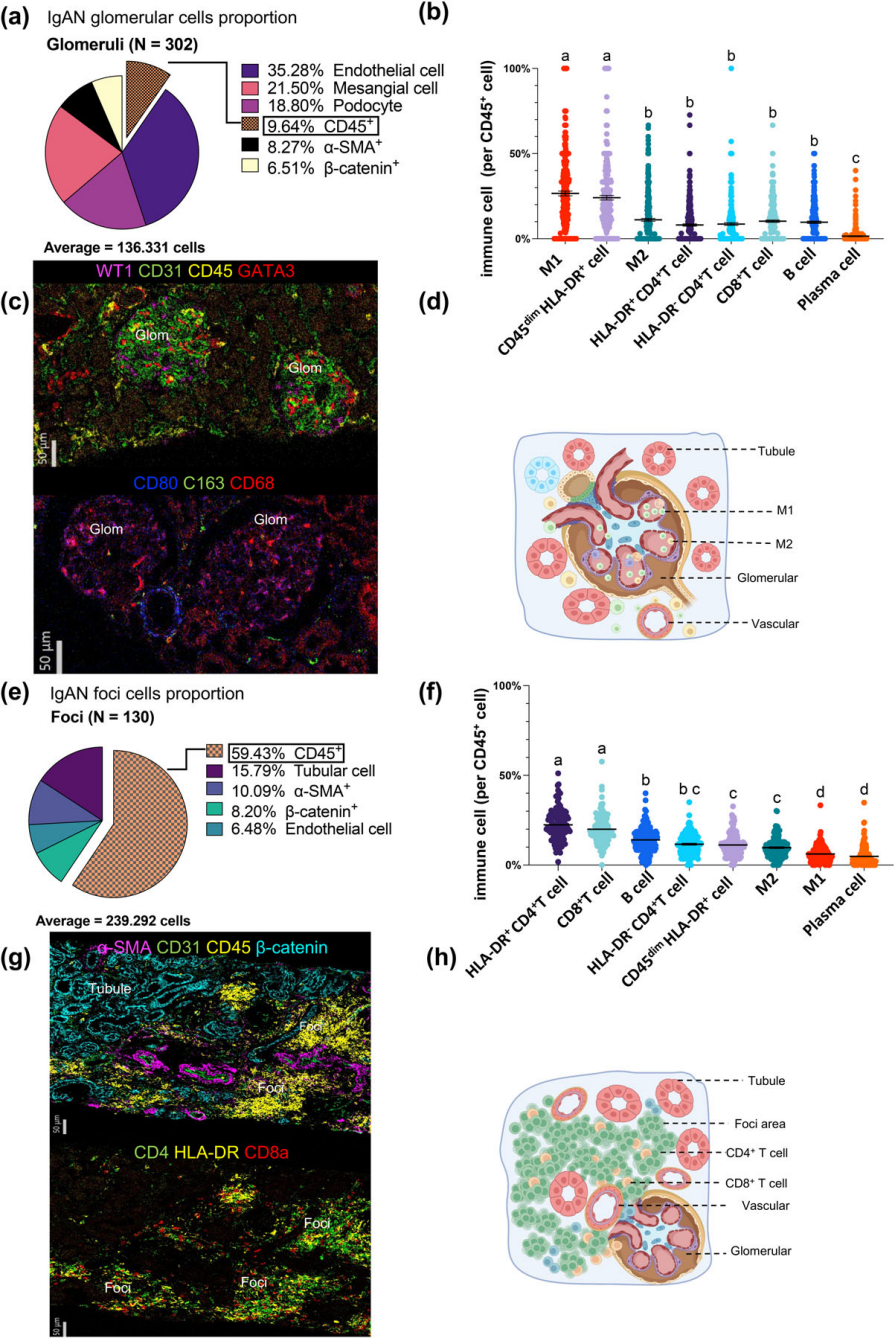
The different immune cell infiltration patterns in glomeruli and foci of IgAN. **(a)** The average cell population percentage in all glomeruli is as follows: *N* = 302, with an average of 136.331 cells per glomerulus. **(b)** Among the 9.64% of CD45^+^ immune cells in IgAN glomeruli, the most abundant subtype is M1‐like macrophages, followed by CD45^dim^ HLA‐DR^+^ and M2‐like macrophages. **(c)** The upper picture displays the podocytes (WT1, magenta), endothelial cells (CD31, green), mesangial cells (GATA3, red) and immune cells (CD45, yellow) in the glomerular area. The representative picture is from patient 043, diagnosed with Lee III (M1E1S1T0C1). Scale bar = 50 μm. The lower image displays M1‐like macrophages (CD80^+^ blue, CD163^−^ green, CD68^+^ red), M2‐like macrophages (CD163^+^, green), bar = 50 μm. **(d)** The simulation diagram illustrates the anatomical and pathological relationships of the left image **(c)**. **(e)** The average cell population percentage in all foci is as follows: N = 130, with an average of 239.292 cells. **(f)** Among the CD45^+^ immune cells (59.43%), the most abundant subtypes were HLA‐DR^+^ CD4^+^ T cells and CD8^+^ T cells. **(g)** The upper picture displays the low magnification view of the tubulointerstitial area: SMC (α‐SMA, magenta), endothelial cells (CD31, green), immune cells (CD45, yellow) and all tubular cells (β‐catenin, teal), scale bar = 50 μm. The representative picture is from patient 778, diagnosed with Lee IV (M1E1S1T0C1). The lower picture displays the immune cells in the tubulointerstitial area, with CD4^+^ T cells (CD4, green), HLA‐DR^+^ T cells (HLA‐DR, yellow), and CD8^+^ T cells (CD8a, red). The same ROI view corresponds to the aforementioned patient. **(h)** The simulation diagram illustrates the anatomical and pathological relationships of the left image **(g)**. Each symbol represents an individual sample. Statistical analysis was conducted using one‐way ANOVA for comparisons among the groups, followed by *post hoc* LSD analysis for pairwise comparisons. The differences are denoted by letters indicating statistical significance. Groups sharing the same letter suggest no statistically significant difference, while different letters indicate a significant difference. Values are expressed as mean ± SEM, and *p*‐values < 0.05 were considered significant and indicated by letters.

To investigate the properties of immune cells within both glomeruli and foci, we calculated the cell proportions across 302 glomeruli and 130 foci. In glomeruli, endothelial cells constituted the majority (35.28%), followed by podocytes (18.8%) and mesangial cells (21.5%). Immune cells accounted for 9.64% of the population, with the remaining clusters comprising α‐SMA^+^ and β‐catenin^+^ cells (Figure 2a). Among immune cells, classical (M1‐like) macrophages and CD45^dim^ HLA‐DR^+^ cells were the most abundant populations, followed by alternative (M2‐like) macrophages (Figures 2b–d). In contrast, foci exhibited a different cellular composition, with a substantial proportion of tubular cells, endothelial cells and broad immune cells, accounting for 59.43% of the total cell population (Figure 2e). Within the immune cell population of foci, HLA‐DR^+^ CD4^+^ T cells and CD8^+^ T cells were the most prominent infiltrating cells, followed by B cells and HLA‐DR^−^ CD4^+^ T cells (Figures 2f–h). These findings highlight marked spatial differences in immune cell infiltration between glomeruli and foci in IgAN kidneys.

### Distinct glomerular cellular compositions underlie pathological heterogeneity in IgAN

**Figure 3:**
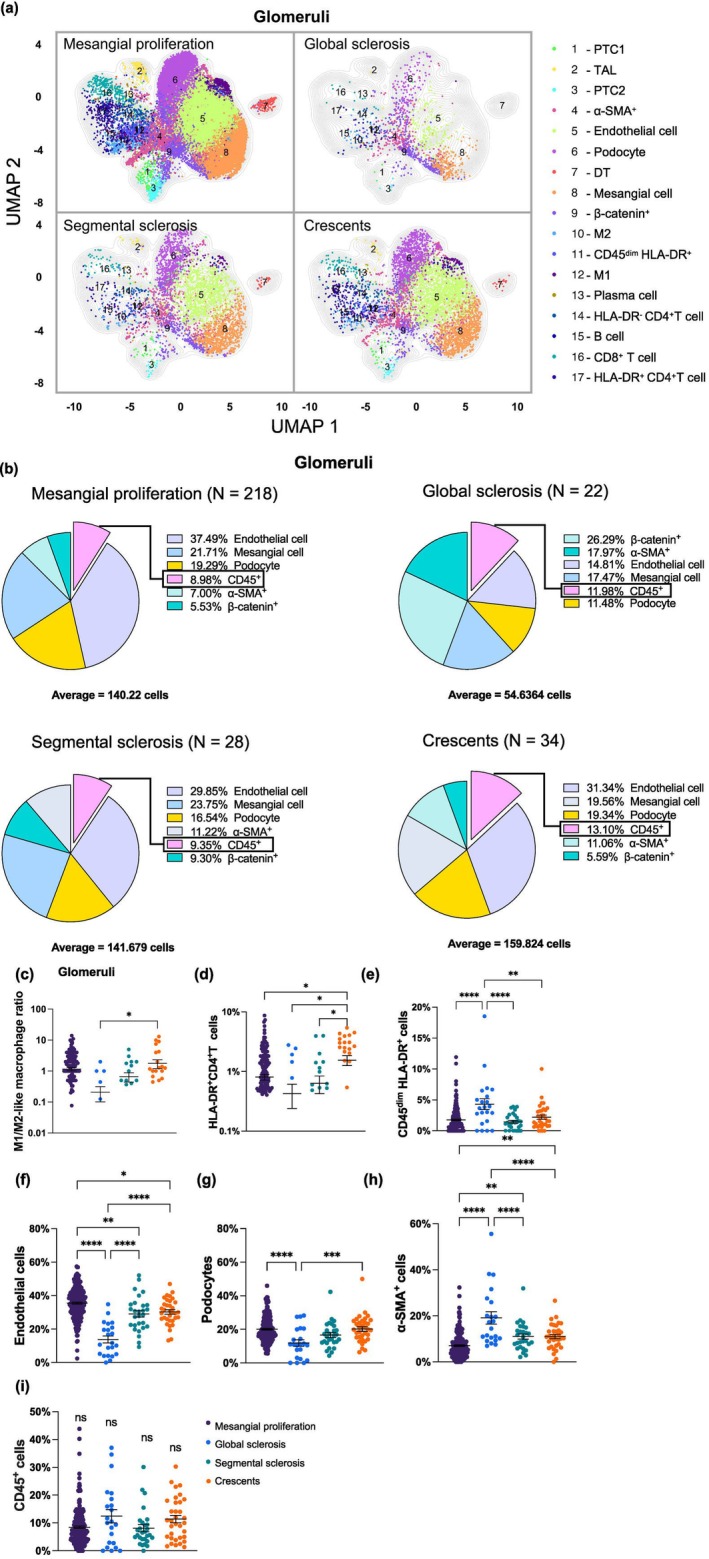
Region‐specific distributions of cells within different pathologically defined glomeruli in IgAN. **(a)** The UMAP illustrates the distribution pattern of cell clusters in all glomeruli as follows: 218 mesangial proliferation, 22 global sclerosis, 28 segmental sclerosis and 34 crescents. **(b)** Pie charts displaying the infiltration of stromal cells and CD45^+^ immune cells in the above four groups. **(c)** The M1/M2‐like macrophage ratio in crescents was the highest among all four subgroups. **(d)** HLA‐DR^+^ CD4^+^ T cells were the highest in the crescents among all four subgroups. **(e)** The CD45^dim^ HLA‐DR^+^ population was elevated in the global sclerosis subgroup. **(f–h)** Endothelial cells and podocytes were decreased significantly in the global sclerosis subgroup, while α‐SMA^+^ clusters were notably increased in the global sclerosis subgroup. **(i)** Proportion of CD45^+^ cells among the four subtypes of glomeruli. Each symbol represents an individual sample (**c–i**). Statistical analysis was conducted using one‐way ANOVA for comparisons among the multiple groups, followed by *post hoc* LSD analysis for pairwise comparisons. Values are expressed as mean ± SEM and *p*‐value (*p < 0.05; **p < 0.01; ***p < 0.001; ****p < 0.0001). ns, non‐significant.

To characterise the heterogeneity of IgAN, we analysed each glomerulus within each patient sample and identified four major subtypes: mesangial proliferation (*N* = 218), global sclerosis (*N* = 22), segmental sclerosis (*N* = 28) and crescents (*N* = 34) by two independent pathologists (Supplementary figure 4e). Global sclerosis represents the most advanced pathological stage, characterised by extensive fibrotic replacement and loss of glomerular architecture. Segmental sclerosis reflects an intermediate stage, in which less than 50% of the glomerular tuft is affected. Crescentic lesions are defined by proliferation of parietal epithelial cells and are associated with pronounced immune activation, whereas mesangial proliferation represents the most common and comparatively mild pathological phenotype in IgAN. Based on these graded levels of severity, UMAP analysis illustrated distinct distributions of glomerular cell populations across the four subtypes (Figure 3a). Glomeruli with mesangial proliferation displayed significant predominance of stromal cells alongside all immune cell populations, accounting for nearly 72% of total glomeruli in the ROIs. In contrast, stromal cell clusters were significantly reduced in glomeruli with global sclerosis, reflecting the predominance of structural damage (Figures 3a and b). Although no significant differences were observed in the overall immune cell populations among the four subtypes (Figure 3i), the proportions of HLA‐DR^+^ CD4^+^ T cells and the M1/M2‐like macrophage ratio were significantly elevated in glomeruli with crescents (Figures 3c and d). This finding aligned with the overt immune activation observed in experimental crescentic glomerulonephritis.^23^ Glomeruli with global sclerosis were characterised by a significant loss of endothelial cells and podocytes, accompanied by an increase in the CD45^dim^ HLA‐DR^+^ population, as well as α‐SMA^+^ cells (Figures 3e–h).

Furthermore, we conducted a comparative analysis of cell signalling intensities. Ki67 and phospho‐STAT3 signalling were found to be most pronounced in crescentic glomeruli, exhibiting a gradual decrease within segmental sclerosis and global sclerosis. In conjunction with an increase in collagen IV deposition, the expression of CD71 and JCHAIN was reduced in global sclerosis (Supplementary figures 3a–f).

### Neighbourhood cell analysis reveals distinct immune interaction patterns across glomerular pathological subtypes

**Figure 4:**
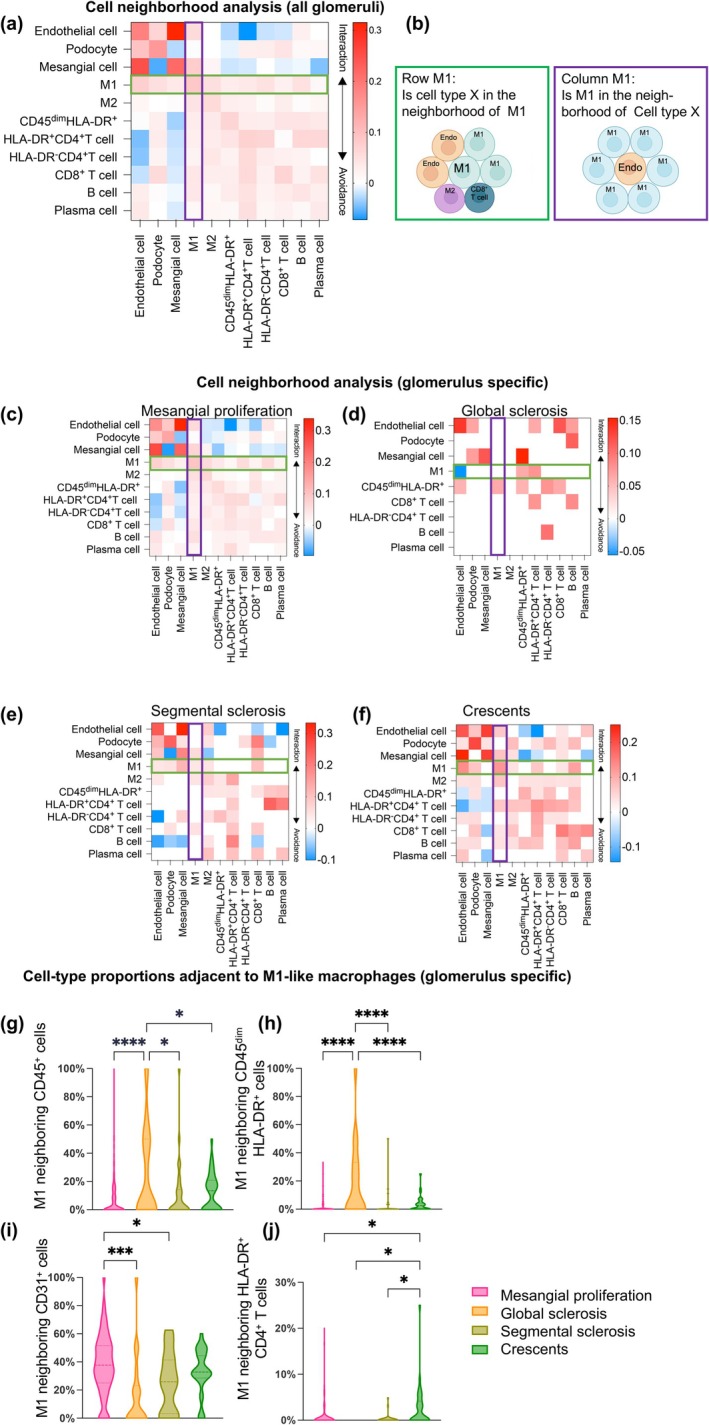
Different patterns of neighbourhood cells with M1‐like macrophages in glomeruli of IgAN. **(a)** All interactions among glomerular cell types are represented as a heatmap, where each row indicates whether the cell type in the row is significantly neighbored (red) or avoided (blue) by the cell type in the column. Rows visualise the significance of all cell types surrounding a given cell type of interest (M1, highlighted in green). Columns visualise the significance of the cell type of interest (M1) surrounding other cell types (highlighted in purple). **(b)** Schematic illustrating the directional aspects of M1‐like macrophages with neighbouring cell types, as visualised in the heatmap. **(c–f)** Heatmaps depict the cell interactions in four glomerular types. Red arrows indicate significant interactions, while blue arrows indicate avoidance. Heatmap colour intensity represents the correlation coefficient. **(g–j)** For each of the four glomerular groups, the proportions of each kind of cell neighbouring M1‐like macrophages, relative to all neighbouring cells, were statistically compared. We analysed CD45^+^ cells, including CD45^dim^ HLA‐DR^+^ cells, which exhibited the most significant spatial proximity to M1‐like macrophages in global sclerotic glomeruli. The proximity or interaction between M1‐like macrophages and endothelial cells was most pronounced in glomeruli with mesangial proliferation. Finally, interactions between HLA‐DR^+^ CD4^+^ T cells and M1‐like macrophages were most prominent in crescentic glomeruli. Statistical analysis was conducted using one‐way ANOVA for comparisons among the multiple groups, followed by *post hoc* LSD analysis for pairwise comparisons. Values are expressed as mean ± SEM and *p*‐value (*p < 0.05; ***p < 0.001; ****p < 0.0001).

To systematically investigate the spatial interactions between stromal and immune cells that underlie disease progression, we conducted a neighbourhood interaction analysis. Significant associations or avoidances between pairs of cell‐type were measured based on their spatial proximity across all glomeruli (Figure 4a). The right‐hand interaction matrix depicts the neighbouring relationships, with green rows representing cell types adjacent to the target cells (*e.g*. M1) and purple columns indicating the target cells (*e.g*. M1) in proximity to other cell types (Figure 4b). Given the prominent role of macrophages in IgAN glomeruli, we explored the interaction patterns of M1‐like macrophages across different glomerular subtypes (Figures 4c–f). In glomeruli with mesangial proliferation, the crosstalk between endothelial cells and M1‐like macrophages emerged as a main event (Figure 4c). In contrast, in glomeruli with global sclerosis, immune cells, particularly CD45^dim^ HLA‐DR^+^ cells, were the primary interacting partners of M1‐like macrophages, reflecting a significant loss of stromal cells and persistent immune cell activity (Figure 4d). In segmental sclerosis, a pivotal interaction was identified between mesangial cells, CD8^+^ T cells and M1‐like macrophages (Figure 4e). The interaction between stromal cells, T/B cells, and M1‐like macrophages were observed in glomeruli with crescents (Figure 4f).

To further quantify these patterns, we compared the proportions of immune cells among the neighbouring cells surrounding M1‐like macrophages across the four glomerular subtypes. Interestingly, in global sclerosis glomeruli, immune cells occupied a larger proportion among neighbouring M1‐like macrophages, predominantly composed of CD45^dim^ HLA‐DR^+^ cells, showing statistical significance (Figures 4g and h). In pure mesangial proliferative glomeruli, the neighbouring cells of M1‐like macrophages remained endothelial cells (Figure 4i), while in crescentic glomeruli, the predominant neighbouring cells were HLA‐DR^+^ CD4^+^ T cells (Figure 4j). Collectively, these findings reveal subtype‐specific patterns of macrophage‐centred cellular interactions, underscoring the dynamic and heterogeneous immune microenvironments within IgAN glomeruli.

### In‐depth analysis of immune cell correlations with clinical manifestations in IgAN

**Figure 5:**
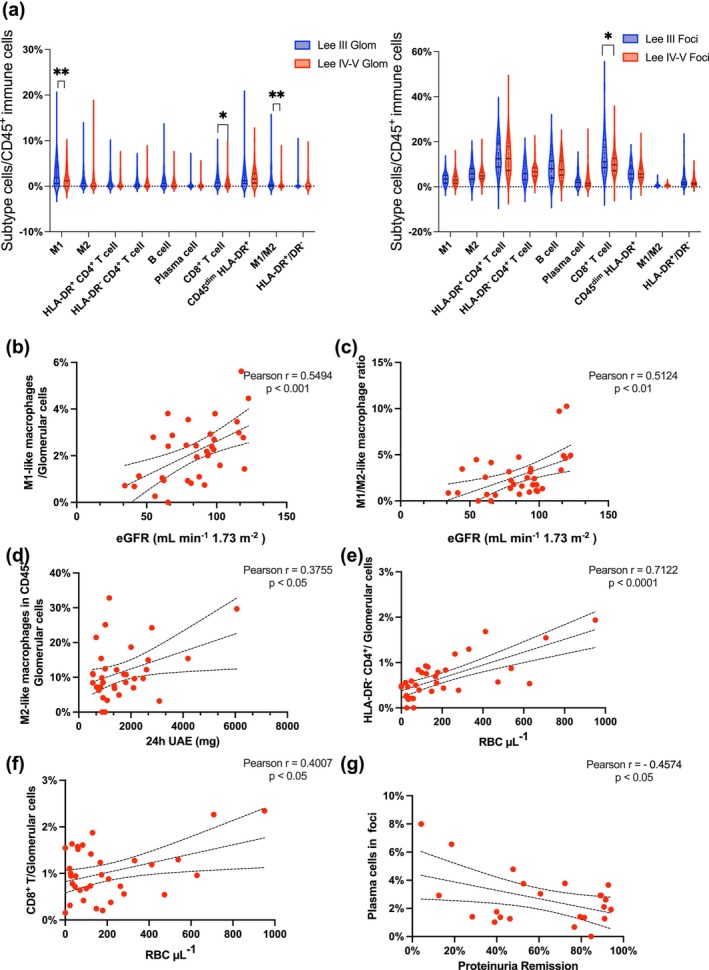
The correlation of clinical manifestation with immune cells in IgAN. **(a)** The proportion of immune cells in glomeruli and foci varies across Lee grades. Lee III (*N* = 21) and Lee IV–V (*N* = 13). Glomerular M1‐like macrophages and the M1/M2‐like macrophage ratio are significantly decreased in Lee IV–V, while CD8^+^ T cell abundance is decreased in Lee IV–V in both glomerular and foci. **(b, c)** M1‐like and M1/M2‐like macrophage ratio in glomeruli are positively correlated with eGFR in this cohort (*N* = 34). **(d)** The proportion of M2‐like macrophages in CD45^+^ cells was positively correlated with 24‐h UAE. **(e, f)** HLA‐DR^−^CD4^+^ T cells and CD8^+^ T cells in glomeruli were positively correlated with haematuria levels. **(g)** The percentage of plasma cells in the foci was negatively correlated with proteinuria reduction percentage after 1 year of treatment. Data are expressed as mean ± SEM, with Student's two‐tailed *t*‐test *p < 0.05; **p < 0.01. Pearson correlation analysis was used for normally distributed data. UAE: urinary albumin excretion; RBC: red blood cell; eGFR: estimated glomerular filtration rate.

To assess the clinical utility of IMC‐derived cellular profiles, we combined our findings with corresponding IgAN Lee grade and clinical parameters. Significant differences in immune cell proportions were observed within both glomeruli and focal regions across disease severities (Figure 5a). Specifically, the proportion of M1‐like macrophages and the M1/M2‐like macrophage ratio decreased significantly in the glomeruli of patients with severe IgAN (Lee grades IV–V) compared with those with mild IgAN (Lee grade III). In parallel, CD8^+^ T cells exhibited features consistent with exhaustion in both glomerular and focal regions as disease severity increased. Notably, within the mesangium, CD71 levels were elevated in severe IgAN (Lee grades IV–V), while JCHAIN expression showed a corresponding decrease (Supplementary figures 3g and h).

We next calculated the average proportion of each cell cluster across ROIs for each patient. The percentage of M1‐like macrophages in glomeruli exhibited a positive correlation with the estimated glomerular filtration rate (eGFR), with a Pearson's correlation coefficient of *r* = 0.5494 (Figure 5b). Similarly, the M1/M2‐like macrophage ratio showed a positive correlation with eGFR, with a Pearson's correlation coefficient of *r* = 0.5124 (Figure 5c). The proportion of M2‐like macrophages within the CD45^+^ cell population positively correlated with 24‐h UAE (urinary albumin excretion), with a Pearson correlation coefficient of *r* = 0.3755 (Figure 5d). Regarding haematuria levels, HLA‐DR^−^ CD4^+^ T cells and CD8^+^ T cells were positively correlated with RBC μL^−1^, with Pearson correlation coefficients of *r* = 0.7122 and 0.4007, respectively (Figures 5e and f). During follow‐up, all patients received regular treatment with ACEi/ARB, either alone or in combination with glucocorticoids. Proteinuria levels stabilised at approximately 0.5 g day^−1^ after treatment (Supplementary figure 1b). Interestingly, the percentage reduction in proteinuria at 1 year was inversely correlated with the plasma cell proportion in the foci, with a Pearson correlation coefficient of *r* = −0.4574 (Figure 5g).

## Discussion

In this study, we present the first characterisation of immune cell populations and cell–cell interactions within human IgAN using IMC. IMC is a powerful technology that integrates multiplexed imaging with mass spectrometry through laser ablation of metal‐labelled antibodies. Since its introduction, IMC has been successfully applied in kidney research. Singh *et al*. pioneered the characterisation of the two‐dimensional landscape of the human kidney using IMC with 23 protein markers in 2019.^24^ In 2023, Louis *et al*. reported on the application of IMC in lupus nephritis (LN), comparing class II and IV LN.^25^ Asowata *et al*. utilised kidneys from deceased transplant organ donors as a human kidney disease model in 2024.^26^ More recently, Kondo *et al*. reported on the use of IMC in diabetic kidney disease (DKD), comparing stage II and III DKD in 2024.^27^ Building upon the above findings, we initially applied IMC to investigate the immune landscape of IgAN patients and translate our findings on 17 cell populations into clinically relevant insights.

When comparing our data with previous IMC studies of kidneys, particularly the work by Singh *et al*., we observed that although their antibody panel included 23 markers, only 10 cell populations were identified. This discrepancy is primarily because of their distinct research focus and antibody selection, as their panel contained a substantial number of tubular cell markers. While Singh *et al*. analysed normal kidney tissue, our panel was specifically designed to focus on glomerular structures and immune cells, incorporating several cell signalling markers associated with IgAN.^21^ Accordingly, we deliberately reduced the number of tubular cell markers and expanded a broader range of immune cell markers, making direct data comparison with other IMC data challenging. Notably, that study reported that immune cells account for approximately 5% of all cells in normal kidney tissue. In contrast, our results showed that immune cells comprise roughly 10% of all cells within the glomerular compartment and nearly 50% within the interstitial compartments of IgAN kidneys.

The advantage of IMC lies in its ability to define immune cell populations with greater detail and spatial resolution, thereby overcoming limitations of previous single‐cell RNA sequencing studies in which glomeruli and tubules were pooled during tissue dissociation.^11^, ^12^, ^13^, ^28^ In our cell proportion analysis, we observed predominant infiltration of M1‐like macrophages in the glomeruli, with secondary infiltration of M2‐like macrophages. This aligned with prior cohort studies (*N* = 621) on glomerular macrophages (CD68^+^, CD206^+^), suggesting their role in determining the efficacy of immunosuppressive treatments in IgAN.^29^ Our findings, supported by IMC, specifically indicated the critical role of glomerular macrophages in IgAN pathogenesis. Past studies have highlighted the enrichment of M2‐like macrophages in the tubulointerstitial region of IgAN,^30^ consistent with their established association with fibrosis. In contrast, M1‐like macrophages are well‐recognised as a pro‐inflammatory macrophage subtype,^31^ typically polarised during the early phase of the disease and serving as the primary phagocytes for efferocytosis.^32^ Nevertheless, the precise contribution of M1‐like macrophages to IgAN progression remains to be fully elucidated. One plausible mechanistic explanation is that M1‐like macrophages exert stage‐dependent functions in IgAN, contributing to early immune surveillance and clearance of immune complexes within glomeruli, while their decline in advanced disease reflects a transition toward chronic fibrosis‐dominated pathology.

Among the four glomerular types, our findings indicated that the M1/M2‐like macrophage ratio and the proportion of HLA‐DR^+^ CD4^+^ T cells were lowest in sclerotic glomeruli and highest in crescentic glomeruli, indicating heightened immune activity in crescentic glomeruli, consistent with the understanding that crescentic formation often necessitates aggressive immunosuppressive treatment.^23^ Moreover, Ki‐67 intensity and phospho‐STAT3 intensity data further supported this pattern, indicating the highest proliferation events in the crescentic glomeruli. Although the overall CD45^+^ immune cell population showed no significant difference, the decrease in M1‐like macrophage polarisation and CD4^+^ T cell activation in sclerotic glomeruli may correspond with an increase in the proportion of other immune cells, such as CD45^dim^ HLA‐DR^+^ cells. Furthermore, we leveraged the spatial resolution of IMC for cell neighbourhood analysis using histoCAT on glomeruli with varying pathologies, an analysis that had not been previously interpreted in IMC kidney data. Our findings revealed prominent endothelial cells with M1‐like macrophage interactions in mesangial proliferation glomeruli. Notably, this kind of interaction was absent in sclerotic glomeruli; instead, M1‐like macrophages with immune cells, such as CD45^dim^ HLA‐DR^+^, exhibited more prevalent interactions. Together, these findings support a mechanistic model in which glomerular immune microenvironments evolve dynamically during IgAN progression, with early macrophage–endothelial interactions giving way to macrophage–lymphocyte dominated niches that promote crescent formation and irreversible structural damage. This spatially resolved immune remodelling provides a potential explanation for the heterogeneous pathological manifestations of IgAN and their variable responses to immunosuppressive therapy.

Beyond the pathological analysis of immune infiltrates, we conducted a detailed correlation analysis between individual cell types and clinical phenotypes. We observed that the M1/M2‐like macrophage ratio was significantly decreased in severe IgAN and positively correlated with eGFR, suggesting that the macrophages expressing pro‐inflammatory M1‐like markers could serve as a potential indicator for early treatment timing in IgAN. Regarding adaptive immune cell infiltration, T cells' abundance was positively correlated with the levels of haematuria in the glomeruli. This finding supports the link between haematuria and immune activity with adverse kidney outcomes,^33^ indicating that treatment for massive haematuria may necessitate T‐cell inhibition. Finally, in our follow‐up study, greater post‐treatment reduction in proteinuria was associated with lower plasma cell infiltration in the foci prior to medication. This finding suggests that plasma cell infiltration may be a chronic and detrimental factor in IgAN, highlighting the potential benefit of anti‐plasma cell therapies, such as CD38‐targeting antibodies. Importantly, our findings are derived from a retrospective, observational cohort study; while this design enables identification of clinically relevant associations, it does not establish causality or demonstrate therapeutic efficacy.

Overall, the results of this study lay the groundwork for a deeper understanding of immunophenotypes with clinical and pathological heterogeneity in IgAN. Nevertheless, several limitations inherent to the application of IMC in kidney tissue analysis should be acknowledged. First, the antibody panel lacked complement proteins. Although IMC can only detect intracellular complement components, secreted complement proteins are known to play a crucial role in the pathogenesis of IgAN. Second, the inclusion of healthy controls was not feasible because of the challenges of obtaining renal biopsy samples from healthy donors. Therefore, we conducted a within‐group comparison based on Lee grade‐based grouping and morphological subtypes. In addition, our findings primarily reflect the immune landscape of untreated IgAN. The lack of longitudinal data capturing immune cell dynamics following interventions such as ACEi/ARB or immunosuppressive therapies limits the broader clinical applicability of our results. Our findings suggest that glucocorticoid therapy may be particularly relevant in patients whose biopsy samples exhibit higher densities of T cells or macrophages within the glomerular compartment. However, this hypothesis requires validation in prospective clinical studies. Future investigations incorporating longitudinal sampling and treatment response assessment will be essential to fully elucidate the dynamic immune microenvironment of IgAN and its clinical implications.

## Conclusion

Our findings demonstrate that distinct glomerular immune cell compositions and spatial interaction patterns are closely associated with clinical and pathological heterogeneity in IgAN. Spatially resolved immune phenotypes identified by IMC may provide a framework for patient stratification and guide the development of personalised immunomodulatory treatment strategies.

## Methods

### Human renal biopsy samples and clinical data acquisition

Inclusion Criteria: (a) Disease Diagnosis: Primary IgAN. (b) Patients under 65 years of age. (c) Proteinuria: Protein excretion ≥ 0.5 g day^−1^. (d) Haematuria: Presence of varying degrees of haematuria. (e) Renal Function: eGFR ≥ 30 mL min^−1^ 1.73 m^−2^. Exclusion Criteria: Secondary IgAN and specific types of IgAN such as Minimal Change Disease, Focal Segmental Glomerulosclerosis, and crescentic glomerulonephritis.

The renal biopsies were performed at the time these patients were diagnosed with primary glomerular nephritis between 2023 and 2024. Samples were processed as routine FFPE specimens (immersion in 10% neutral buffered formalin followed by paraffin embedding). Sections were cut at 5 μm and stored at 4°C. FFPE renal biopsy samples diagnosed as IgAN based on pathological evaluation were included in this study. All procedures were conducted after institutional review board approval (The First Affiliated Hospital of Xiamen University Ethics Committee, 2024 Scientific Research Ethics Approval [No. 177]). Clinical data, including follow‐up data up to 2 years post‐treatment, were collected retrospectively through chart review (Supplementary table 1 and Figure 1).

### Antibody conjugation

We performed conjugation of metal isotopes (Fluidigm, California, USA) with primary antibodies (Abcam, Cambridge, UK) following established protocols^34^ when pre‐conjugated metal‐labelled antibodies were unavailable from Fluidigm. In this study, we evaluated the panel of 35 proteins (Table 1 and Supplementary figure 2) based on two criteria: (a) their expression in human tissue as determined by our previous experiments^35^ and (b) their performance according to published IMC literature.^24^, ^27^ All primary antibodies underwent validation studies for specificity and performance using kidney FFPE samples through immunofluorescence prior to experimentation. Antibodies that yielded unsatisfactory results were replaced until a satisfactory antibody was identified.

**Table 1:** Overview of antibody panel.

Serial no.	Metal‐Tag	Antigen	Target	Supplier	Clone (validated)	Dilution
1	141	α‐SMA	Smooth muscle cells	Fluidigm	1A4	1:200
2	142	CD20	B cells	Fluidigm	SP32	1:100
3	143	VIM	Podocytes, endothelial cells, mesangium, fibroblasts	Fluidigm	D21H3	1:400
4	144	JCHAIN	Plasma cells	abcam	EPR23130‐113	1:100
5	145	CD80	Macrophages M1	abcam	EPR1157 (2)	1:100
6	146	WT1	Podocytes	abcam	CAN‐R9(IHC)‐56–2	1:100
7	147	CD163	Macrophages M2	Fluidigm	EDHU‐1	1:100
8	148	ICOS	Tfh cells	Fluidigm	D1K2T	1:100
9	149	phospho‐STAT3	Phenotypic events	abcam	phospho Y705	1:100
10	150	CD19	B cells	abcam	SP291	1:100
11	151	CD31	Endothelial cells	abcam	JC/70A	1:200
12	152	CD45	Immune cells	Fluidigm	CD45‐2B11	1:200
13	153	FN1	Phenotypic events	abcam	EPR23110‐46	1:200
14	154	CD71	Mesangial cells:transferrin receptor	abcam	EPR20584	1:200
15	155	–	–	–	–	–
16	156	CD4	CD4^+^ T cells	Fluidigm	EPR6855	1:100
17	158	UMOD	TAL	abcam	EPR20071	1:400
18	159	CD68	Macrophages	Fluidigm	KP13	1:100
19	160	HLA‐DR	T cell stimulation	abcam	EPR3692	1:400
20	161	CD56	NK cells	abcam	CAL53	1:100
21	162	CD8a	CD8^+^ T cells	Fluidigm	C8/144B	1:200
22	163	CXCR5	Tfh cells	abcam	EPR23463‐30	1:100
23	164	CD45RO	Activated T cells	abcam	UCH‐L1	1:200
24	165	LRP2	PT	abcam	EPR26093‐84	1:400
25	166	CALB1	DCT	abcam	EP3478	1:400
26	167	β‐catenin	Tubular epithelial cells	abcam	E247	1:400
27	168	Ki67	Phenotypic events	Fluidigm	B56	1:400
28	169	CD161	MAIT	abcam	OTI1D8	1:100
29	170	CD3	T cells	Fluidigm	Polyclonal C‐terminal	1:100
30	171	CD27	B cells	Fluidigm	EPR8569	1:100
31	172	PDGFRβ	Mesangial cells	abcam	EPR26830‐84	1:100
32	173	Col‐IV	Basement membrane	abcam	EPR22911‐127	1:100
33	174	Syndecan‐1/CD138	Plasma cells/Plasmablasts	abcam	EPR6454	1:100
34	175	CD38	Plasma cells/Plasmablasts	abcam	EPR4106	1:100
35	176	GATA3	Mesangial cells	abcam	EPR16651	1:200
36	191	DNA1/2	Nuclear	Fluidigm	cell‐ID intercalator	1:400

CALB1, Calbindin1; DCT, Distal Convoluted Tubule; FN1, Fibronectin; LRP2, MEGALIN; MAIT, Mucosal‐Associated Invariant T cells; PT, Proximal Tubule; TAL, Ascending of Henle loop; Tfh, T follicular helper cells; VIM, VIMENTIN.

### Staining

Tissue sections on slides were baked at 60°C for 25 min and then deparaffinised using two rounds of 100% fresh xylene. Rehydration was performed through sequential washes in 100% ethanol (2×), 95% ethanol (2×), 85% ethanol (1×), 70% ethanol (1×), followed by deionised water. The sections underwent antigen retrieval in Tris‐EDTA buffer (pH 9.0, Abcam, UK) at 98°C for 15 min. Slides were rinsed with deionised water (1×), Dulbecco's Phosphate‐Buffered Saline (DPBS) (1×), and deionised water for 5 min each. To block nonspecific binding, tissues were treated with Superblock Blocking Buffer (Thermo Fisher Scientific, Massachusetts, USA) for 25 min. Subsequently, the antibody cocktail, diluted as specified in Table 1, was applied to the sections and incubated at 4°C overnight. Post‐incubation, slides were washed sequentially with 0.2% Triton X‐100 (2×) and DPBS (2×). Following conjugation, the slides were incubated with Intercalator‐Ir (diluted 1:400 in DPBS) for 30 min at room temperature. Finally, slides were rinsed, air‐dried, and stored at 4°C until ready for ablation.

### ROI selection strategy

ROIs were selected based on the pathological morphology identified by haematoxylin and eosin (HE) staining of renal biopsy tissues. On each slide: (a) 2–8 ROIs (600 μm × 600 μm) were manually delineated to maximise inclusion of glomeruli and adjacent inflammatory infiltrates, thereby reflecting the general pathology of each biopsy (Supplementary figure 4a). The number of ROIs acquired per slide was determined by the actual presence of glomeruli rather than a random selection. (b) Within each selected ROI, all glomeruli (0–5 per ROI) and foci were manually outlined, respectively (Supplementary figures 4c and d), yielding 432 analytical units and ensuring a comprehensive assessment of glomerular and foci lesions.

### Imaging mass cytometry acquisition

The Hyperion imaging mass cytometry System (Fluidigm, California, USA) was calibrated using a three‐element tuning slide following the manufacturer's standard protocol. To ensure successful tuning, a minimum threshold of 1500 mean dual counts for 175 Lu was established. ROIs were ablated at 200 Hz for approximately 60 min. Data were saved as MCD files and visualised using the Fluidigm MCD Viewer. To improve differentiation between antibody signals and background noise, each marker was assessed individually, establishing a minimum signal threshold of 1 or 2 dual counts in the MCD Viewer (refer to Supplementary figures 4a and b and Figure 2).

### Cell segmentation

CellProfiler^36^ was employed to create cell masks and measure marker expression levels. Nuclei were initially detected as primary objects using ilastik‐generated probability maps, followed by cytoplasmic expansion until reaching adjacent cells or the background. These cell masks enabled the identification of individual cells and facilitated the extraction of single‐cell data, including marker intensity, spatial coordinates, and neighbourhood interactions from the original images.

### Data analysis, image visualisation and neighbourhood analysis

Samples and their corresponding masks were loaded into HistoCAT (version 1.76)^37^ to delineate glomerular and foci regions through gating. Post‐gating, samples were exported from HistoCAT as CSV files containing cell ID, marker signal intensities, and spatial coordinates for each cell. Data processing and visualisation were conducted using R and HistoCAT. Cell types were classified using an R‐based implementation of FlowSOM. Unless specified otherwise, raw IMC data were normalised to the 99th percentile and scaled to a range of 0–1.^38^ R packages employed included flowCore, cytofkit, dplyr, Rumap and additional tools. Neighbourhood analysis was performed in R and HistoCAT, with custom scripts used to import cell type data into HistoCAT, applying a 4‐pixel expansion to identify neighbouring cells. All other settings and procedures adhered to established protocols.^37^

### Statistical analysis

Figure legends provide the statistical parameters. Cell populations were quantified as a percentage of a given cell type relative to either the total number of cells or the total number of immune cells identified within each glomerulus and foci. Differences in cell percentage were assessed using the GraphPad Prism 10 software, Student's two‐tailed *t*‐test, or one‐way ANOVA, with a *p*‐value < 0.05 considered significant.

## Author contributions

Conceptualisation, LZ, XC and LL; Methodology, LZ and XXY; Investigation, XXY, JXS, XC, HYZ and LJY; Formal Analysis, LZ and XXY; Writing – Original Draft, LZ and XXY; Writing – Review and Editing, LZ and XXY; Funding Acquisition, LZ, XXY; Resources, LZ, ZJL, LPS and XXY; Supervision, LZ, LL and XXY.

## Conflict of interest

The authors declare no conflict of interest.

## Supporting information


Supplementary figure 1

Supplementary table 1

## Data Availability

The data that support the findings of this study are openly available in Zenodo at https://doi.org/10.5281/zenodo.15878164, reference number 15878164.
