# Supplementary material for: Unveiling the immune landscape and microenvironmental architecture of IgA nephropathy using single‐cell imaging mass cytometry
Source: Clin Transl Immunology. 2026 Mar 13;15(3):e70088. doi: 10.1002/cti2.70088 (PMC13093778; doi:10.1002/cti2.70088)
Supplement: Supplementary file 1 — Supplementary figure 1 [file CTI2-15-e70088-s001.pdf]

Supplementary figure 1

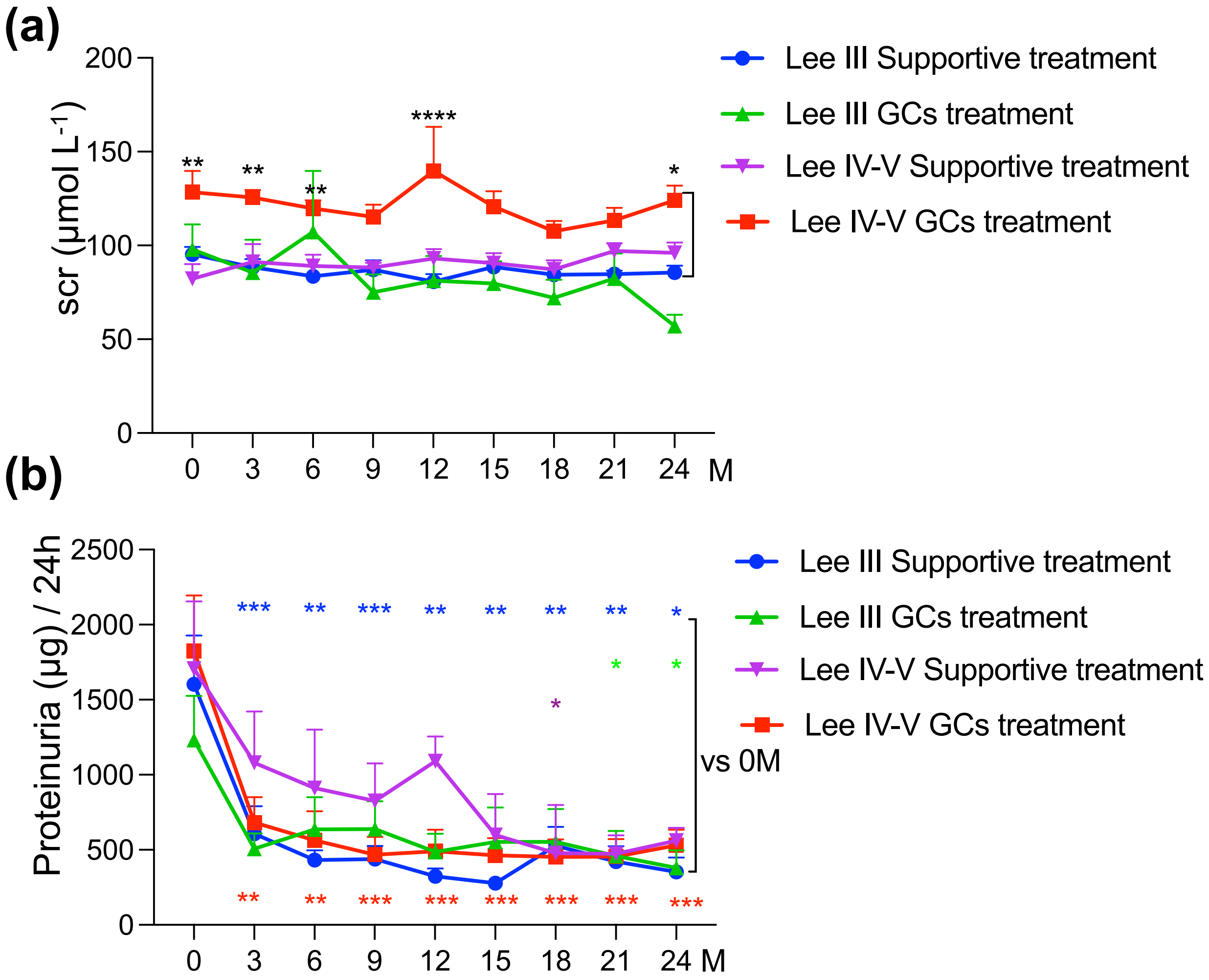

Supplementary figure 1. Patient follow-up data and treatment effects. **(a)** Twenty-four–month follow-up of serum creatinine (scr). **(b)** Twenty-four–month follow-up of 24-hour proteinuria. Data are expressed as mean ± SEM. Two-way ANOVA with Dunnett's multiple comparisons test was used. * $p < 0.05$, ** $p < 0.01$, **** $p < 0.0001$ for Lee grades IV–V GCs vs. Lee grade III supportive treatment subcategories in (a). * $p < 0.05$, ** $p < 0.01$, *** $p < 0.001$ indicate comparisons of each treatment subgroup with baseline (0 months, before treatment) in (b).

Supplementary figure 2

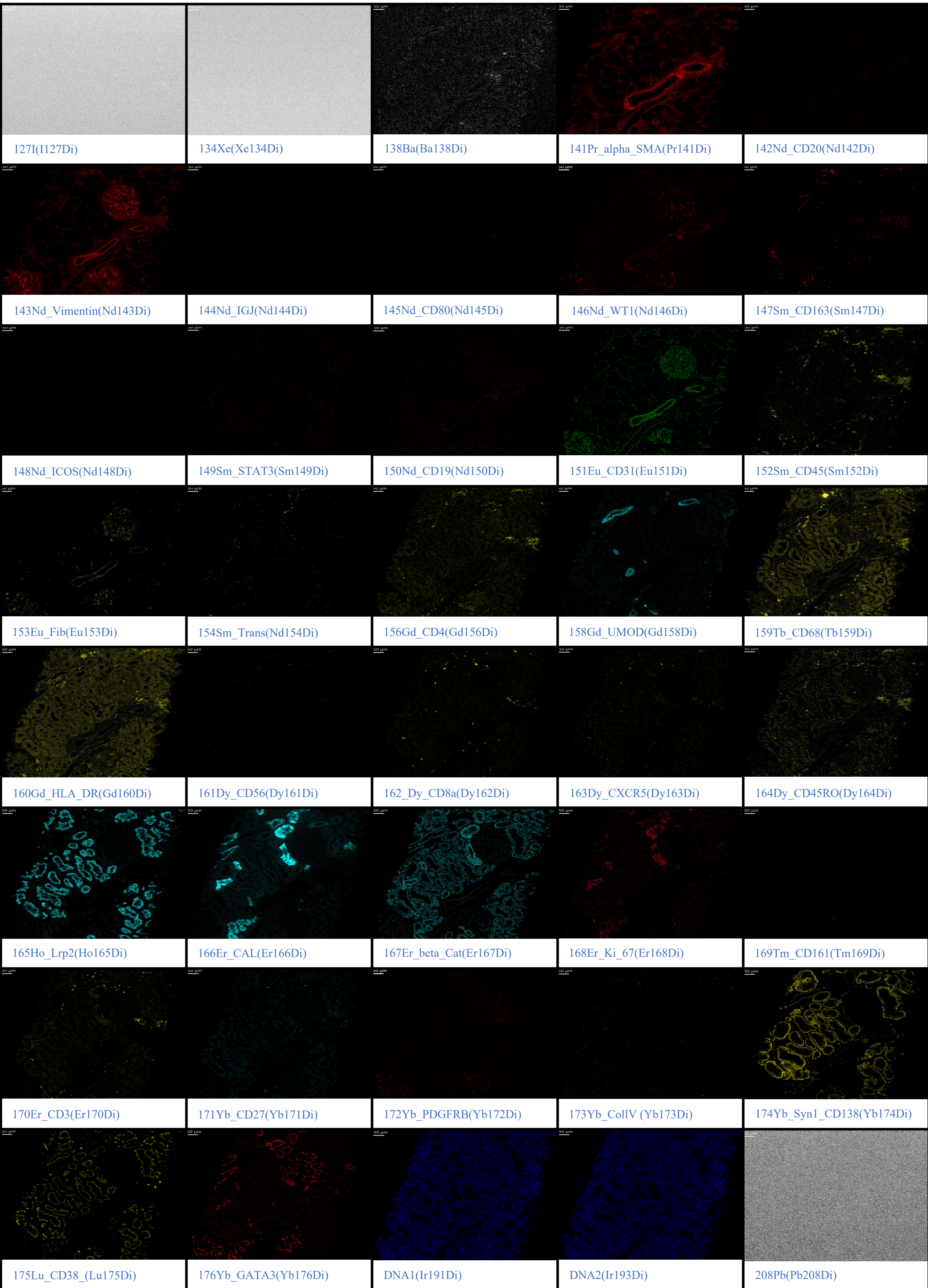

Supplementary figure 2. Staining results for the 35-antibody panel. This figure shows the experimental staining results for all 35 antibodies in tissue from the same patient shown in Supplementary figure 1. Each channel corresponds to one antibody, and the names of the 35 proteins are listed in Table 1. Scale bar = 50 μm. IGJ: JCHAIN; Trans: CD71(Transferrin receptor 1, TfR1); ColIV: Collagen IV.

Supplementary figure 3

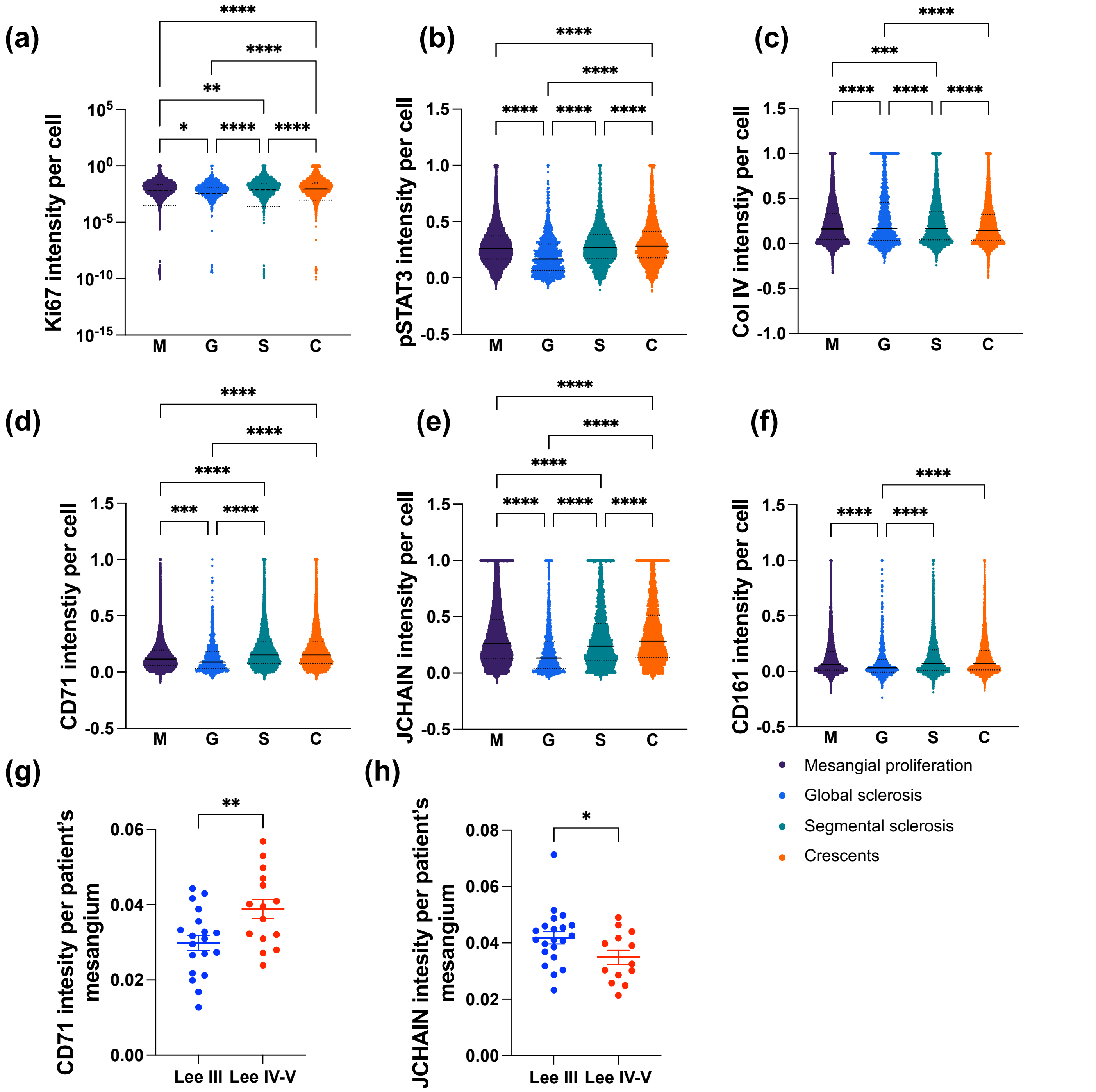

Supplementary figure 3. Cell expression intensity levels among subtypes of IgAN glomeruli. **(a-b)** Ki67 (proliferation marker) and phospho-STAT3 (immune cell activation signal) intensity per cell among the four subtypes of glomeruli. **(c)** Col IV intensity per cell among the four subtypes of glomeruli. **(d-f)** IgAN progression and mechanism-related markers: CD71, JCHAIN and CD161 expression intensity per cell among the four subtypes of glomeruli. N = 31464, 1216, 4111 and 5580 cells, respectively. **(g-h)** CD71 and JCHAIN expression per patient's mesangium between mild and severe Lee grades (N = 21 for Lee grade III, N=13 for Lee grades IV–V). Statistical differences among multiple groups were assessed using one-way ANOVA, while comparisons between two groups were performed using Student's two-tailed *t*-test; Data are presented as mean ± SEM, and *p*-values less than 0.05 were considered significant (* $p < 0.05$; ** $p < 0.01$; *** $p < 0.001$; **** $p < 0.0001$).

Supplementary figure 4

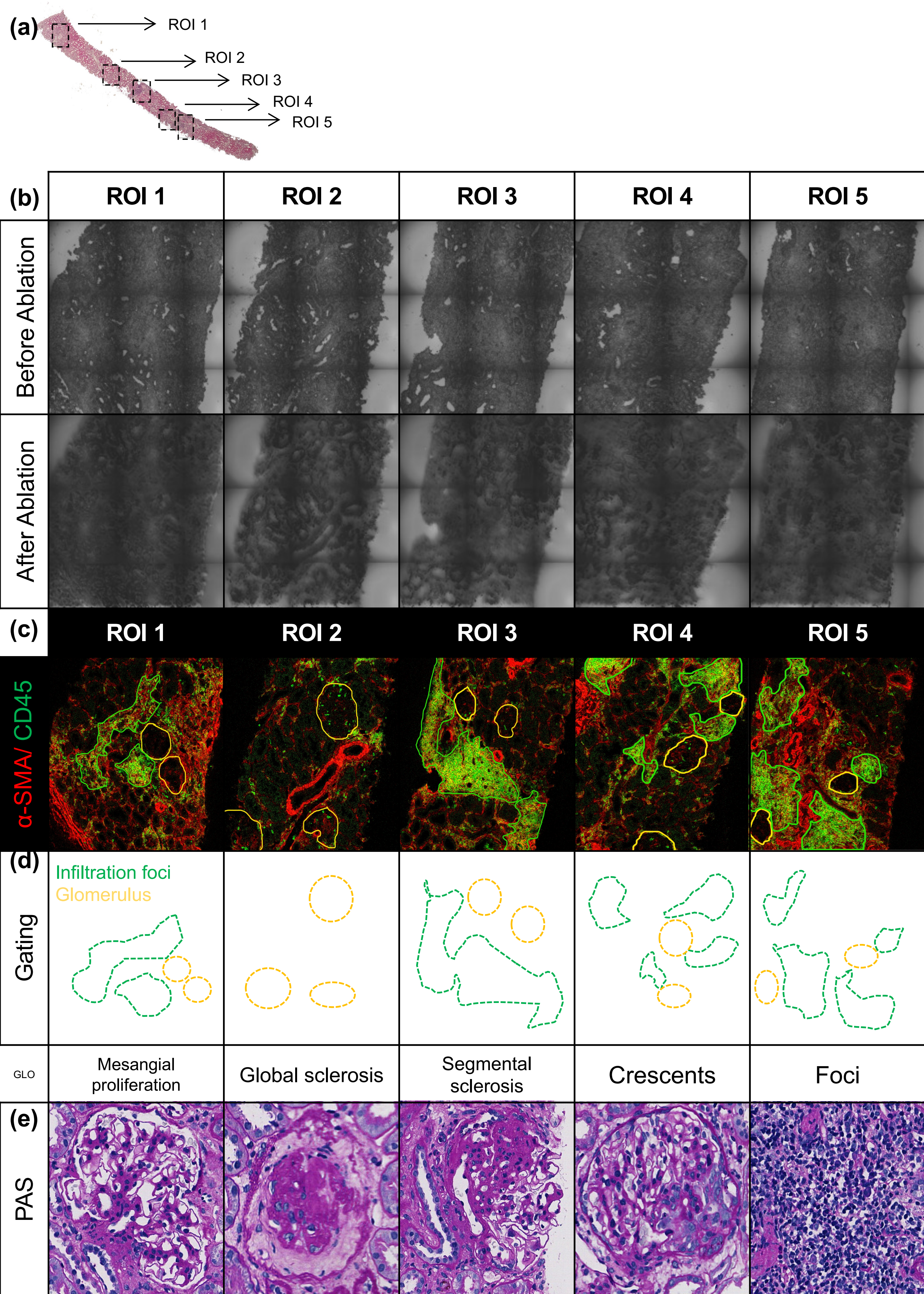

Supplementary figure 4. Workflow for ROI selection and analysis in IgAN kidney biopsy specimens. A representative kidney biopsy specimen is shown for workflow demonstration. For each case, three consecutive slides were obtained: the first slide was stained with HE or PAS for pathological assessment and localization, and the other slides were used for experiments. **(a)** The upper panel shows the HE-stained section under low magnification (5x), with ROIs identified and marked by squares. **(b)** In the corresponding experimental section, the same ROIs were located using the instrument window based on the previous mentioned slide, and tissue within the ROIs was isolated by laser. The images show the tissue before and after laser dissection as observed**. (c)** For each ROI, the positions of glomeruli and interstitial infiltrates were further confirmed using α-SMA and CD45 channel, which were then outlined for subsequent data analysis. The upper image displays the results from the experimental slide. **(d)** The lower image shows the gating strategy. **(e)** The figure also presents the morphological classification of each glomerulus and foci in the pathological section. For analysis on the experimental slide, glomerular classification and subsequent analyses were accurately matched to the pathological markings. HE: hematoxylin and eosin; PAS: Periodic Acid-Schiff stain.
