# Supplementary material for: Unveiling the immune landscape and microenvironmental architecture of IgA nephropathy using single‐cell imaging mass cytometry
Source: Clin Transl Immunology. 2026 Mar 13;15(3):e70088. doi: 10.1002/cti2.70088 (PMC13093778; doi:10.1002/cti2.70088)
Supplement: Supplementary file 2 — Supplementary table 1 [file CTI2-15-e70088-s002.pdf]

Supplementary table 1. Patients information

Serial No.	Sex	Age	Study ID	BP(mmHg)	Urine RBC(/uL)	24h UAE(mg/day)	Scr(μmol/L)	eGFR(mL/min/1.73 m²)	Clinical Diagnosis	Others	Pathological ID	Pathological Diagnosis	ROI number
1	F	32	cx1	normal	2169.5	1356	79	85.33	nephritis syndrome	-	261	IgAN，Lee Ⅳ（M1 E0 S1 T0 C1）	7
2	F	36	cx2	normal	172.8	1727	141	41.18	nephritis syndrome	-	038	IgAN，Lee Ⅳ-Ⅴ（M1 E0 S1 T1 C1）	4
3	M	32	cx3	normal	49.7	669	130	62.2	nephritis syndrome	-	962	IgAN，Lee Ⅳ（M1 E0 S1 T2 C1）	6
4	M	42	cx4	normal	70.3	4189	200	34.44	nephritis syndrome	-	961	IgAN Lee Ⅳ（M1 E1 S1 T2 C1）	5
5	M	40	cx5	normal	473.5	1814	99	81.73	nephritis syndrome	-	050	IgAN，Lee Ⅳ（M1 E0 S1 T0 C2）	4
6	F	49	cx6	normal	20.5	1173	86	68.34	proteinuria	HTD	257	IgAN，Lee Ⅳ（M1 E0 S1 T0 C0）	6
7	M	28	cx7	normal	87.0	2658	112	79.7	nephritis syndrome	-	435	IgAN，Lee Ⅳ（M1 E1 S1 T1 C1）	6
8	M	36	cx8	148/89	46.2	538	147	54.7	nephritis syndrome	HTD	396	IgAN，Lee Ⅳ（M1 E0 S1 T0 C1）	7
9	F	27	cx9	normal	330.8	1021	81	85.75	nephritis syndrome	-	778	IgAN，Lee Ⅳ（M1 E1 S1 T0 C1）	5
10	F	37	cx10	normal	263	1544	101	61.21	nephritis syndrome	-	970	IgAN，Lee Ⅳ（M1 E0 S1 T1 C1）	8
11	F	32	cx11	144/90	27.2	1009	112	55.95	nephritis syndrome	HTD	209	IgAN，Lee Ⅳ-Ⅴ（M1 E1 S1 T2 C0）	4
12	F	45	cx12	normal	538.8	2596	63	102.39	nephritis syndrome	-	051	IgAN，Lee Ⅳ（M1 E1 S1 T0 C2）	6
13	F	34	cx13	normal	412.4	3091	134	44.42	nephritis syndrome	-	207	IgAN，Lee Ⅳ（M1 E0 S1 T1 C1）	3
14	F	28	cx14	normal	951	2471	72	98.18	nephritis syndrome	-	398	IgAN，Lee Ⅲ（M1 E0 S1 T0 C1）	7
15	M	46	cx15	normal	100	1453	98	79.33	nephritis syndrome	-	980	IgAN，Lee Ⅲ（M1 E0 S0 T0 C1）	6
16	M	33	cx16	144/92	25.8	1800	73	115.85	proteinuria	HTD	306	IgAN，Lee Ⅲ（M1 E0 S1 T0 C1）	5
17	F	32	cx17	normal	178.7	1092	54	119.9	nephritis syndrome	-	266	IgAN，Lee Ⅲ（M1 E0 S1 T0 C1）	2
18	M	55	cx18	normal	628.6	548	94	78.32	nephritis syndrome	-	420	IgAN，Lee Ⅲ（M1 E0 S1 T0 C1）	4
19	F	35	cx19	normal	166.7	2804	97	65.19	nephritis syndrome	Hyperuricemia	717	IgAN，Lee Ⅲ（M1 E0 S1 T0 C1）	4
20	M	33	cx20	normal	280.5	536	124	65.39	nephritis syndrome	OSAHS	879	IgAN，Lee Ⅲ（M1 E0 S1 T0 C1）	6
21	F	32	cx21	156/85	normal	1009	72	95.46	proteinuria	HTD	490	IgAN，Lee Ⅲ（M1 E0 S1 T0 C1）	6
22	M	45	cx22	normal	60.7	902	116	65.15	nephritis syndrome	Gout	868	IgAN，Lee Ⅲ（M1 E0 S1 T0 C1）	3
23	M	27	cx23	144/96	130.3	931	93	96.58	nephritis syndrome	HTD	049	IgAN，Lee Ⅲ（M1 E0 S1 T0 C1）	4
24	F	30	cx24	normal	34.2	890	55	122.7	nephritis syndrome	-	021	IgAN，Lee Ⅲ(M1 E1 S0 T0 C0)	5
25	F	29	cx25	normal	148.7	930	63	114.57	nephritis syndrome	-	155	IgAN，Lee Ⅲ（M1 E0 S1 T0 C1）	3
26	M	27	cx26	normal	58.4	2006	92	97.85	nephritis syndrome	-	783	IgAN，Lee Ⅲ（M1 E1 S1 T0 C1）	5
27	F	33	cx27	normal	708.6	774	56	117.64	nephritis syndrome	-	615	IgAN，Lee Ⅲ（M1 E1 S1 T0 C1）	7
28	M	20	cx28	149/93	82.9	6060	95	98.87	nephritis syndrome	HTD	928	IgAN，Lee Ⅲ（M1 E1 S1 T0 C0）	4
29	M	33	cx29	normal	normal	2140	94	91.41	proteinuria	-	634	IgAN，Lee Ⅲ（M1 E0 S0 T0 C0）	6
30	M	32	cx30	normal	31.8	2098	98	87.53	nephritis syndrome	-	043	IgAN，Lee Ⅲ（M1 E1 S1 T0 C1）	6
31	M	40	cx31	normal	22.4	858	89	92.96	nephritis syndrome	HTD	526	IgAN，Lee Ⅲ（M1 E0 S1 T0 C1）	5
32	F	33	cx32	normal	119.6	833	54	119.06	nephritis syndrome	-	524	IgAN，Lee Ⅲ（M1 E0 S1 T0 C1）	3
33	F	25	cx33	normal	207	687	76	93.92	nephritis syndrome	-	686	IgAN，Lee Ⅲ（M1 E1 S1 T0 C1）	4
34	M	24	cx34	150/82	122.3	1373	97	93.74	nephritis syndrome	HTD	684	IgAN，Lee Ⅲ（M1 E0 S1 T0 C1）	5

BP: Blood pressure. RBC: Red blood cell. UAE: Urine albumin excretion. Scr: Serum creatinine. eGFR: estimated Glomerular Filtration Rate. ROI: regions of interest. HTD: Hypertension. OSAHS:Obstructive Sleep Apnea-Hypopnea Syndrome.

Lee Ⅲ: Diffuse mesangial proliferation and thickening with focal and segmental variation, occasional small crescents and adhesions, with focal interstitial edema and infiltrate occaionally present, Tubular atrophy rare.

Lee Ⅳ: Marked diffuse mesangial proliferation and sclerosis, cresents present in up to 45% of glomeruli, with tubular atrophy, interstitial inflammation, and occasional interstitial foam cells.

Lee Ⅴ: Glomerular, tubular and interstitial changes similar to Ⅳ, but more severe. Crescents present in more than 45% of glomeruli.

Accoding to Oxford Classification of IgA nephropathy 2016: M means “Mesangial hypercellularity” (Mesangial score should be assessed in periodic acid-Schiff-stained sections. If more than half the glomeruli have more than three cells in a mesangial area, this is categorized as M1, if not M0); E means “Endocapillary hypercellularity”(if present E1, if not E0); S means “Segmental glomerulosclerosis with or without the presence of an adhesion” (if present S1, if not S0); T means “Tubular atrophy/interstitial fibrosis” (according to percentage of cortical area involved: 0-25% T0, 26-50% T1, ＞50% T2); C means “cellular and/or fibrocellular cresents” (C0: no crescents, C1: crescent in a least 1 glomerulus and ＜25% glomeruli, C2: crescents in at least 25% of glomeruli).

Study ID: identification number for clinical data report; Pathological ID: pathologicla identification number; ROI number: the number of ROI get from the sample.
